# *In vitro* Development of Chemotherapy and Targeted Therapy Drug-Resistant Cancer Cell Lines: A Practical Guide with Case Studies

**DOI:** 10.3389/fonc.2014.00040

**Published:** 2014-03-06

**Authors:** Martina McDermott, Alex J. Eustace, Steven Busschots, Laura Breen, John Crown, Martin Clynes, Norma O’Donovan, Britta Stordal

**Affiliations:** ^1^National Institute for Cellular Biotechnology, Dublin City University, Dublin, Ireland; ^2^Department of Drug Discovery and Biomedical Sciences, South Carolina College of Pharmacy, University of South Carolina, Columbia, SC, USA; ^3^Department of Medical Oncology, Beaumont Hospital, Royal College of Surgeons in Ireland, Dublin, Ireland; ^4^Department of Histopathology, St James’ Hospital, Trinity College Dublin, Dublin, Ireland; ^5^Department of Medical Oncology, St Vincent’s University Hospital, Dublin, Ireland

**Keywords:** chemotherapy, cancer, drug-resistance, cell lines, selection strategy

## Abstract

The development of a drug-resistant cell line can take from 3 to 18 months. However, little is published on the methodology of this development process. This article will discuss key decisions to be made prior to starting resistant cell line development; the choice of parent cell line, dose of selecting agent, treatment interval, and optimizing the dose of drug for the parent cell line. Clinically relevant drug-resistant cell lines are developed by mimicking the conditions cancer patients experience during chemotherapy and cell lines display between two- and eight-fold resistance compared to their parental cell line. Doses of drug administered are low, and a pulsed treatment strategy is often used where the cells recover in drug-free media. High-level laboratory models are developed with the aim of understanding potential mechanisms of resistance to chemotherapy agents. Doses of drug are higher and escalated over time. It is common to have difficulty developing stable clinically relevant drug-resistant cell lines. A comparative selection strategy of multiple cell lines or multiple chemotherapeutic agents mitigates this risk and gives insight into which agents or type of cell line develops resistance easily. Successful selection strategies from our research are presented. Pulsed-selection produced platinum or taxane-resistant large cell lung cancer (H1299 and H460) and temozolomide-resistant melanoma (Malme-3M and HT144) cell lines. Continuous selection produced a lapatinib-resistant breast cancer cell line (HCC1954). Techniques for maintaining drug-resistant cell lines are outlined including; maintaining cells with chemotherapy, pulse treating with chemotherapy, or returning to master drug-resistant stocks. The heterogeneity of drug-resistant models produced from the same parent cell line with the same chemotherapy agent is explored with reference to P-glycoprotein. Heterogeneity in drug-resistant cell lines reflects the heterogeneity that can occur in clinical drug resistance.

## Introduction and Historical Perspective

The development of chemotherapy drug-resistant cancer cell lines is a long established approach for investigating the mechanisms of cytotoxicity and resistance to chemotherapy agents. One of the first publications to describe the development of an anti-cancer drug-resistant *in vitro* model, which exhibited acquired resistance to a chemotherapy drug, was published in 1970 (1). Resistant cell lines were developed from parental Chinese hamster cells using a stepwise increase in treatment dose with actinomycin D. This induced 2500-fold greater resistance to the drug than that observed in the parental cells. These resistant cell lines were also cross resistant to other chemotherapy drugs such as vinblastine and daunorubicin. Some earlier drug-resistant cell lines were developed in the 1950 and 1960s using *in vivo* mouse models, including models resistant to methotrexate (2, 3), vinblastine, terephthalanilide (4), and the guanine analog, 8-azaguanine (5).

Publications in this research field usually place little emphasis on how the drug-resistant cell lines were established in the laboratory. The development of drug-resistant cell lines can take anything from 3 to 18 months in the laboratory and many decisions are taken along this journey. This review summarizes the major methodological approaches for developing drug-resistant cell lines *in vitro* with reference to the literature and includes several case studies from our experience.

### IC_50_ values and fold resistance

Drug-resistant cell models are developed in the laboratory by repeatedly exposing cancer cells growing in cell culture to drugs. The surviving daughter resistant cells are then compared to the parental sensitive cells using combination cell viability/proliferation assays such as the MTT (6), acid phosphatase (6), or clonogenic assays (7). The sensitivity of these paired cell lines is usually determined by exposing them to a range of drug concentrations and then assessing cell viability. The IC_50_ (drug concentration causing 50% growth inhibition) for these paired cell lines can be used to determine the increase in resistance known as fold resistance by the following equation:

Fold Resistance=IC50 of Resistant Cell Line∕IC50 of Parental Cell Line

## What is a Clinically Relevant Level of Resistance?

To determine the level of drug resistance that occurs in the clinical treatment of cancer we can compare cell lines that have been established from cancer patients before and after chemotherapy (Table 1) (8–14). The majority of cell lines listed in Table 1 developed from patients post-chemotherapy show a two- to five-fold increase in resistance to the agents the patients were treated with, based on a comparison of IC_50_ values. Three cell lines had higher levels of resistance but these were still relatively low-level at ~8–12-fold higher than the parental cells (PEO4, SK-3, and GLC-16).

**Table 1:** **Cell lines established from cancer patients before and after chemotherapy**.

Cancer type	Parent cell line (established)	Chemotherapy received	Resistant cell line (established)	Fold resistance to chemotherapy received	Reference
Lung	EBC-2 (18th September 1997)	CIS, IFO, VIND	EBC-2/R (4th October 1997)	CIS – 2.3, IFO^a^ – 3.2, VIND – 0.77	(8)
	SK-1 (August 1986)	CYC, ADR, ETO, VINC, RAD	SK-2 (March 1987)	ADR – 1.2, ETO – 1.2, CYC^b^ – 1.3	(10)
		CIS, ETO	SK-3 (May 1987)	CIS – 8.6, ETO – 6.2	
	TM1 (April 1987)	CYC, ADR, ETO, VINC	TM2 (September 1987)	CYC^b^ – 5.4, ADR – 3.0, ETO – 3.5	
	GLC-14 (December 1984)	CYC, DOX, ETO	GLC-16 (October 1985)	DOX – 3.18, ETO – 12.1	(11)
Neuroblastoma	KP-N-AY (October 1984)	ADR, CIS, CYC, VINC	KP-N-AYR (December 1985)	ADR – 3.0, CIS – 2.7	(9)
Ovarian	PEO1 (February 1982)	CIS, CHL, 5-FU	PEO4 (November 1982)	CIS – 8.72	(12, 13)
		CIS, CHL, 5-FU	PEO6 (February 1983)	CIS – 4.64	(12, 13)
	PEA1	CIS, PRED	PEA2	CIS – 4.30	(13, 14)
	PEO14	CIS, CHL	PEO23	CIS – 4.48	(13, 14)

*ADR, adriamycin; CIS, cisplatin; CHL, chlorambucil; CYC, cyclophosphamide; DOX, doxorubicin; ETO, etoposide; 5-FU, fluorouracil; IFO, ifosfamide; RAD, radiation; PRED, prednimustine; VINC, vincristine; VIND, vindesine*.

*^a^Used 4-hydroperoxy ifosfamide (the active form of ifosfamide)*.

*^b^Used 4-hydroperoxycyclophosphamide (the active form of cyclophosphamide)*.

### Clinically relevant vs. high-level laboratory models

For the purposes of this review we will divide drug-resistant cell models into two categories: clinically relevant models or high-level laboratory models. Both types of models have their advantages and disadvantages for research.

Clinically relevant models are developed with the aim of trying to mimic the conditions cancer patients experience during chemotherapy. Doses of drug are lower, and a pulsed treatment strategy is often used where the cells recover in drug-free media. This mimics the cycles of chemotherapy a patient receives in the clinic. Disadvantages to clinically relevant models can include unstable resistance, very low-level resistance, and small molecular changes to detect and analyze. Based on the cell lines derived from the patients before and after chemotherapy shown in Table 1; we have defined clinically relevant resistance as a two- to five-fold increase from the IC_50_ value of the parent cell line. Examples of clinically relevant models are shown in Table 2 (15–21).

**Table 2:** **Different selection strategies and classification of resulting drug-resistant cell lines**.

Cancer type	Parent cell line	Selecting agent	Exposure	Dose	Population	Resistant cell line	Fold resistance to selecting agent	Development time (months)	Classification	Reference
Cervical	KB-3-1	CIS	Continuous	Stepwise and	Whole	KBCP10	1152	UNK	High-level lab	(22, 23)
		COL		mutagenesis	Cloned	KB-8-5-11	40	UNK	High-level lab	(24)
Leukemia	CCRF-CEM	EPI	Pulse	Constant	Whole	CEM/E25	7	UNK	Clinically relevant	(15, 16)
			Continuous	Stepwise	Whole	CEM/E1000	94	8 from E25	High-level lab	
	K562	DNR	Pulse	Stepwise	Whole	K562/DNR	3	2	Clinically relevant	(17)
Lung	DLKP	ADR	Continuous	Stepwise	Whole	DLKP-A	322	18	High-level lab	(25)
	A549	PAC	Pulse	Constant	Whole	A549-txl	5.5	2.5	Clinically relevant	(18)
	SKLU1					SKLU1-txl	5.0		Clinically relevant	
	SKMES1	PAC				SKMES1-txl	24.7		High-level lab	
		DOCE				SKMES1-Txt	29.1		High-level lab	
	DMS53	PAC				DMS53-txl	6.3		Clinically relevant	
		DOCE				DMS53-Txt	1.8		Clinically relevant	
	DLRP	DOCE				DLRP-Txt	4.1		Clinically relevant	
	H69	CIS	Pulse	Constant	Whole	H69CIS200	1.5–2	8	Clinically relevant	(19, 20)
		OX				H69OX400	
Ovarian	IGROV-1	CIS	Pulse	Stepwise	Whole	IGROVCDDP	8.41		Clinically relevant	(21)

*ADR, adriamycin; CIS, cisplatin; DNR, daunorubicin; DOCE, docetaxel; EPI, epirubicin; OX, oxaliplatin; UNK, unknown*.

High-level laboratory models are developed with the aim of understanding potential mechanisms of toxicity and resistance to chemotherapy agents. Doses of drug are often high and treatment doses are escalated over time. Cells are frequently grown continually in the presence of drug or highly drug-resistant clones are selected from a mixed population. In some earlier drug-resistant models, mutagenesis was also induced prior to drug treatment (22, 23). High-level models are often more stably resistant and therefore easier to maintain in culture for an ongoing research project. Levels of resistance are often higher and as such molecular changes associated with the mechanism of resistance are larger and easier to identify. The disadvantage of these models is the higher the level of resistance the less relevant the model becomes to the clinic. Examples of high-level laboratory models are shown in Table 2 (15, 16, 18, 22–25).

## Planning a Selection Strategy for Drug-Resistant Cell Lines

### Choice of parental cell line

Choosing a parental cell line is very important as it is the basis of all the subsequent experiments. The parental cell line should be very easy to maintain in cell culture as resistant variants usually become more challenging to grow. Ideally, the researchers performing the drug-resistant selection in the laboratory should be very familiar with growing the parental cells. Researchers experienced in growing a particular cell line will have more of an idea of when the cells need to be subcultured and when it is best to leave them. This experience is important when deciding when to subculture cells recovering from the drug treatment.

It is also important to consider the patient from whom the cell line is derived. If possible, it is good to choose a chemotherapy and radiation naïve cell line. Previous treatment with chemotherapeutic agents and radiation may have already caused changes in resistance pathways, and increased expression of drug resistance markers that may not be relevant to the agent being studied. However, chemotherapy and radiation naïve cell lines are relatively rare. As an alternative to a chemotherapy naïve cell line, choose a cell line with a relatively low baseline IC_50_ value for the drug of interest as a two- to five-fold increase in resistance will result in an IC_50_ of the daughter resistant cell line remaining within the clinically relevant range. Table 3 shows the clinical characteristics of some commonly used ovarian cancer cell lines as an example of the kind of information that is available for cell lines [(12, 14, 26–38); Sikic, personal communication]. In the case of ovarian cancer, the majority of cell lines commonly used in research are derived from metastatic ascites, and are not chemonaïve (Table 3).

**Table 3:** **Clinical characteristics of ovarian tumors from which ovarian cell lines were established**.

Cell line	Original tumor histology	Isolated from	Treatment received pre-isolation	Response	Reference
59M	Endometrioid/clear cell	Ascites	None	N/A	(26)
EFO27	Mucinous	Solid metastasis	None	N/A	(27)
ES2	Serous/clear cell	Primary tumor	None	N/A	[(28); Sikic, personal communication]
FUOV1	Serous	Primary tumor	None	N/A	(29)
HEY	Serous	Peritoneal deposit and xenograft	Radiotherapy, radium	CR	(26, 30)
HOC1	Serous	Ascites	MEL, CIS, ADR, CYC	PR, PR	(31, 39)
HOC8	Serous	Ascites	MEL	PR	(32, 33)
IGROV-1	Endometrioid/clear cell	Primary tumor	None	N/A	(34)
OAW28	Adenocarcinoma	Ascites	CIS, MEL	NR, NR	(26)
OAW42	Serous	Ascites	CIS	CR	(26)
OC316	Serous	Ascites	CIS, ETO, CYC, TAX	PD, SD	(35)
OVCAR3	Serous	Ascites	CYC, CIS, DOX	Unknown	(26, 36, 37)
PEA1	Adenocarcinoma	Pleural effusion	None	N/A	(14)
PEO1	Serous	Ascites	CIS, CHL, 5-FU	CR	(12, 14)
PEO14	Serous	Ascites	None	N/A	(14)
SKOV3	Adenocarcinoma	Ascites	THI	Unknown	(26)
SNU251	Endometrioid	Ascites	CYC, ADR, CIS	Unknown	(38)

*ADR, adriamycin; CIS, cisplatin; CHL, chlorambucil; CR, complete response; CYC, cyclophosphamide; DOX, doxorubicin; ETO, etoposide; MEL, melphalan; N/A, not applicable; NR, no response; PD, progressive disease; PR, partial response; TAX, paclitaxel; THI, thiotepa*.

### Exposure to chemotherapy agent

The researcher needs to decide what kind of model they are trying to develop, a clinically relevant model or a high-level laboratory model. A clinically relevant model is informed by data gathered from the clinical administration of drug and usually has minimal escalation of the treatment dose. The sky is the limit for a high-level laboratory model where dose escalation is used extensively to achieve a large fold resistance. However, the solubility of the selecting agent will be final limiting factor in how much drug can be applied to cancer cells. Doses that approach the limit of solubility will not be in the clinically relevant range.

The reality is that most selection strategies start out with a clinically relevant strategy and then are escalated within the clinical range and escalated further again beyond the clinical dose range to make a high-level model. The main reasons for this approach are the stability of the resistance phenotype produced and that the resistance established in the daughter cell line is statistically significant when compared to the parent cell line.

Cell lines are frequently cultured in the presence of antibiotics in many laboratories. When establishing a new drug-resistant model, we recommend not using antibiotics as this does not mimic the clinical situation, cancer patients are not continually treated with antibiotics. Resistance mechanisms produced in the presence of antibiotics may not reflect clinical drug resistance.

#### Pharmacokinetics and drug stability

In order to produce a clinically relevant model of drug resistance, it is important to research how the chemotherapy agent is administered in the clinical treatment of cancer. The amount of chemotherapy administered intravenously (IV) is often expressed in the units milligrams per square meter. These can be converted to micrograms per milliliter or micromolar by consulting pharmacokinetic studies on the drug where the concentration achieved in the bloodstream is measured.

Chemotherapy administered by IV is often given in cycles where the patient receives the drug on a weekly or monthly basis. A pulsed-selection strategy where the cells are treated with drug and then the surviving population are allowed to recover in drug-free media mimics this clinical scenario. Pharmacokinetic studies will give a broad range of doses achieved in the bloodstream, the highest immediately after the bolus of drug is administered to the patient, this then drops over the next hours and days depending on the rate of excretion of the drug. This gives a broad dose range to define the clinical relevance of the dose of drug used in the development of a drug-resistant model. A higher dose for several hours could model the bolus of drug, a lower dose for a several days could model the longer excretion of the drug. Following an intravenous bolus injection of 100 mg/m^2^ cisplatin a peak-plasma level of ~6 μg/mL is reached but this quickly drops to <2 μg/mL after 2 h (40) Clearance of cisplatin from the body is triphasic where the distribution half-life is 13 min, the elimination half-life is 43 min, and the terminal half-life is 5.4 days (41). After 24 h, 25% of the initial cisplatin dose has been eliminated from the body with renal clearance accounting for 90%.

Carboplatin has a similar mechanism of action to cisplatin but needs a 20–40-fold higher dose to exhibit the same cytotoxicity as cisplatin. However, only a 10-fold increase in carboplatin dose is required to reach similar intracellular platinum concentrations (42). After intravenous bolus injection of 375 mg/m^2^ carboplatin peak-plasma levels of ~39 μg/mL are achieved, which drops to 9 μg/mL within 2 h (43). Clearance of carboplatin has a distribution half-life of 22 min, an elimination half-life of 116 min, and a terminal half-life of 5.8 days (44). Clearance of carboplatin from the body is primarily by the urine as unchanged drug. After 24 h, 90% clearance is achieved. Carboplatin does not have significant excretion from the renal tubules as seen for cisplatin, instead the glomerular filtrate accounts for the vast majority of elimination. For this reason, glomerular filtration rate (GFR) is linearly related to total renal clearance giving relatively simple pharmacokinetics for carboplatin. Even at high doses evidence suggests that carboplatin has linear pharmacokinetics (45). A formula called the “Calvert formula” has been derived, which is based on the GFR and is used to provide a suitable dose for patients in relation to an area under concentration time curve (AUC) value. AUC is the ratio of the amount of drug that reaches the systemic circulation and the clearance of the drug, which correlates to its clinical efficiency and toxicity. This formula has been validated in a perspective study (46). Conventional doses of carboplatin administered to patients generally are aimed at giving an AUC value of between 5 and 7 mg/mL/min.

The amount of chemotherapy administered orally is usually expressed in the unit milligrams per day. Again pharmacokinetic studies can be used to convert this to a concentration in the bloodstream. A continuous treatment strategy where the cells are cultured constantly in the presence of drug can be clinically relevant for an oral drug given daily or twice daily as a relatively constant amount of the drug is present. Olaparib is a member of the poly (ADP-ribose) polymerase (PARP) inhibitor class of drugs and is administered orally. The maximum tolerated dose of olaparib is 400 mg twice daily. Absorption is rapid and its peak-plasma concentration is reached within 1–3 h. Plasma levels then decline biphasically and it has a terminal elimination half-life of ~5–7 h (47). A phase 1 study on Japanese patients found that peak-plasma values for a single dose of 400 mg olaparib was ~7 μg/mL, which dropped below 0.1 μg/mL after 50 h. For a dose of 400 mg administered twice daily for 15 days, peak-plasma concentrations were found to be similar. The half-life of olaparib was recorded to be between 7 and 11 h across doses ranging from 100 to 400 mg (48).

The chemical stability of drugs used in establishing drug-resistant cell line models is also an important consideration when designing a selection strategy. For example, temozolomide an alkylating agent used in the treatment of glioblastoma and metastatic melanoma when in its active state, has a half-life of 25 and 60 min for the first and second phases (49) whilst docetaxel a microtubule destabilizing agent has a half-life of 12 h (50). Lapatinib, a dual EGFR HER2 inhibitor used in the treatment of HER2-positive breast cancer has a half-life of 24 h (51) whilst the monoclonal HER2 antibody trastuzumab also used in HER2-positive breast cancer has a half-life of over 5 days (52). Drugs with a shorter half-life will have to be dealt with carefully to ensure that cancer cells receive the maximal benefit from drug dosing. Also drugs with a long half-life should be removed from cells long before the models are to be used in experiments. This ensures that residual drug will not remain in the cells and effect proliferation assays comparing survival between the parental and resistant cells.

#### Optimization of treatment dose in parental cell line

**Figure 1:**
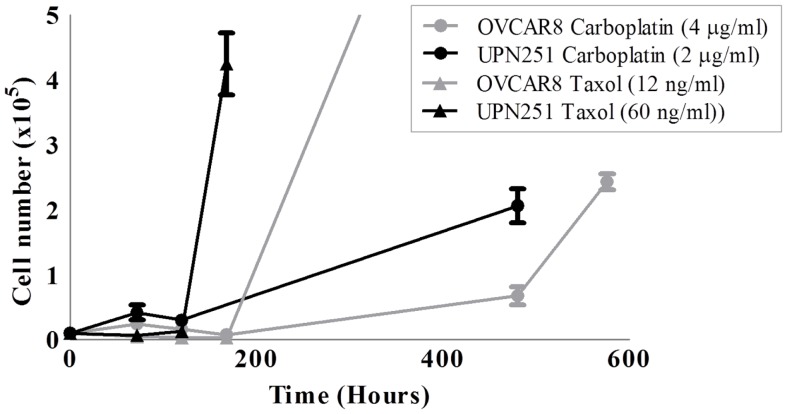
**Recovery of OVCAR8 and UPN251 from carboplatin or paclitaxel treatment**. 1 × 10^4^ cells were treated for 3 days with the indicated doses of either carboplatin or paclitaxel and recovery monitored as described in Section “Methods.”

The dose of drug used must be optimized for the parental cell line selected for use in developing the resistant model. A cytotoxicity assay in the parental cell line can be used to determine a suitable dose range. This dose range can then be compared to the pharmacokinetic information for the drug of interest. The rate of recovery from drug treatment is just as important as the IC_50_; as the rate of recovery can be different between agents even if an equivalently cytotoxic dose is administered to cells. Figure 1 shows the recovery of two ovarian cancer cell lines (OVCAR8 and UPN251) from equivalently cytotoxic doses of carboplatin and paclitaxel. The recovery from paclitaxel is much faster than carboplatin.

The chemotherapeutic drug paclitaxel is frequently given at a dose of 175 mg/m^2^ as a single agent (53, 54). Pharmacokinetic studies for this dose show peak-plasma concentrations as high as 10,000 ng/mL but drop off quickly after 24 h to 50 ng/mL and below (55, 56). In the development of platinum/taxane-resistant OVCAR8 and UPN251 ovarian cancer cells, treatment doses were chosen trailed over the range of IC_20_–IC_80_, and were consistent with doses used in the clinical setting. Paclitaxel doses tested for OVCAR8 and UPN251 were from a range of 2.3–14 and 10–100 ng/mL. Carboplatin doses tested for OVCAR8 and UPN251 were 2.3–18.5 and 0.7–2 μg/mL, respectively. The final chosen doses of paclitaxel and carboplatin displayed an initially large percentage cell death or growth inhibition compared to a control grown in drug-free media. Carboplatin doses of 4 and 2 μg/mL and paclitaxel doses of 12 and 60 ng/mL were chosen for OVCAR8 and UPN251, respectively (Figure 1). After treatment with the selected doses and removal of the drug, the cells were able to return to logarithmic growth ensuring the selection of resistant cell sub populations.

In the development of platinum-resistant H69 small cell lung cancer (SCLC) cells, treatment doses were chosen in the range of IC_10_–IC_40_, and were consistent with doses used in the clinical setting (19). Two exposure times and doses were used for cisplatin and oxaliplatin reflecting differing pharmacokinetic phases of the administration of platinum drugs; 2-h treatments at 1–8 μg/mL and 4-day treatments at 0.2–1.6 μg/mL. The lowest drug concentration treatments all produced 20–30% cell death and growth arrest in H69 cells. Drug-treated cells increased in size and did not aggregate in typical SCLC clumping morphology. Surviving cultures were then retreated when their normal growth rate and clumping morphology had returned, ~3–4 weeks later.

### Population dynamics

In most selection strategies the whole population of cells remains as one group throughout the selection, no cloning or other separation methods are used. If a pulse of drug is given, a small percentage of cells remain, which repopulates the flask. This new population of cells is then retreated with the next pulse (18, 19). Alternatively a low-level of drug is present continuously, the cells adapt to growing in the presence of the drug and then the dose of drug is slowly increased (25).

It is well known that tumors are heterogeneous (57–59). Consequently, the cancer cell lines derived from tumors are also heterogeneous. For example, breast tumors from patients who are BRCA1/2 carriers have been shown to be heterogeneous, where not all cells have lost the second BRCA1/2 allele (60). Selection with chemotherapy agents therefore often result in the isolation of a cell population that already exists in the culture. Indeed, this has been demonstrated for many drug-resistant models, particularly in projects, which examine cancer stem cells (CSCs). CSCs are thought to be responsible for tumor regeneration after chemotherapy. Drug-resistant cell lines are often enriched for markers of stem cells. The stem-cell marker CD133 was found to be enriched in a panel of cisplatin-resistant lung cancer cell lines, with a 5-fold increase in both A549CisR and MORCisR, and a 12-fold increase in H460CisR cells (61).

There are other physical methods of separation available to select different populations from a cell line such as limited dilution or cell sorting by flow cytometry. This can isolate cells which may be more resistant to chemotherapy than other populations within the same cell line (62). The advantage of clonal populations as drug-resistant models for is that there is no drug treatment is required and the resulting model is more stable. The disadvantages however are that many clones must be established and there is no guarantee that the clonal populations derived will display any difference in drug resistance.

Clonal populations can be established by limited dilution. This relies on the ability of the cells to grow independently of each other, and as such may not be suitable for all cell lines. It involves seeding cells at a very low density to result in one cell per well of a 96-well plate. Once the cells grow to confluency, they can be tested as a clonal population. Another method to obtain clonal populations is cloning rings (62). Standard toxicity testing on the clonal populations generated will show whether they display an inherent resistance to the agent of interest.

A combination of drug treatment and cloning of cells has also been used to produce resistant models. KB-8-5-11 colchicine-resistant cells were developed from parental KB-3-1 cells by selecting clones after three stepwise increases in colchicine drug treatment (24). Clones 8, 5, and 11 were the successful clones picked each round of the selection strategy.

Cloning can also be used to investigate heterogeneity within a developed drug-resistant model. A human colon cancer cell line (LoVo) was treated with cisplatin using a continuous exposure ranging from 0.005 to 20.0 μg/mL over 20 months in culture (63). At the end of the treatment, two morphologically distinct subpopulations were observed; these were then cloned by limiting dilution. The subclones showed different patterns of cross resistance to chemotherapy agents. The first clone overexpressed the ABC efflux transporter P-glycoprotein (P-gp) and the other clone did not. Heterogeneity was also seen in cisplatin-resistant models developed from a human pancreatic cancer cell line with a mutation in DNA repair protein BRCA2. Fourteen cisplatin-resistant clones were obtained. In 7 of 14 clones, the functionality of BRCA2 had been restored by secondary mutations, the remaining clones still had a non-functional BRCA2 protein (64).

### Risk-reduction strategies: Comparative selection

It is reasonably common to have difficulties developing resistance or to produce drug-treated daughter cell lines which have not increased in resistance relative to the parental cell line. Selection strategies which do not produce drug resistance are interesting from a clinical perspective as this is what we want to achieve for cancer patients. Unfortunately, failures to develop drug resistance are generally not reported in the literature.

**Figure 2:**
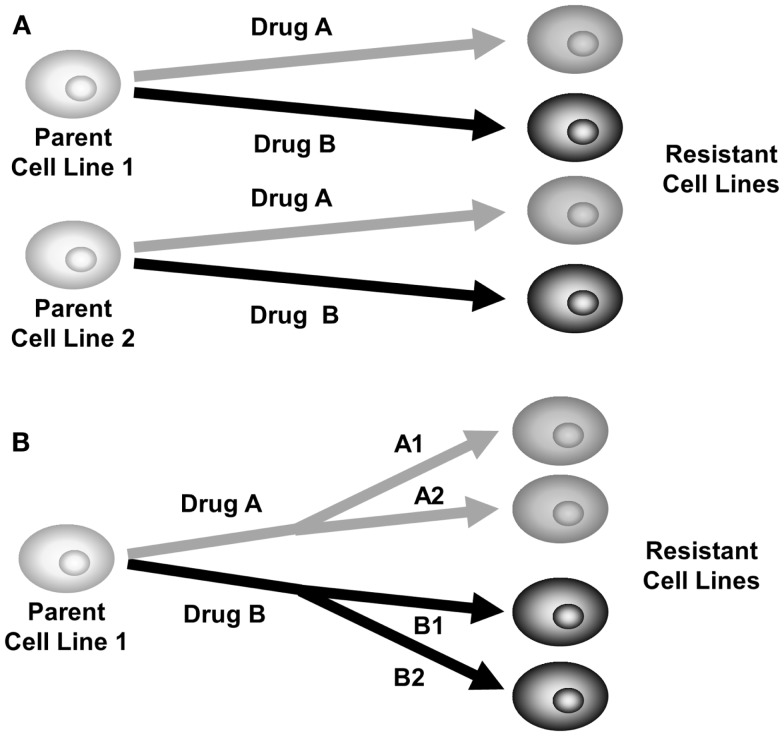
**Comparative selection of drug-resistant cell lines**. **(A)** Plan for selection of two parent cell lines with two different drugs to produce four drug-resistant daughter cell lines. **(B)** Plan for selection of one parent cell line with two drugs at different doses or treatment intervals, producing four drug-resistant daughter cell lines.

As a risk-reduction strategy, a comparative selection strategy should be performed, where selection of multiple cell lines or multiple chemotherapeutic agents are performed in parallel in the laboratory. Figure 2A shows a strategy where two parental cell lines are each treated with two chemotherapy agents, producing four different daughter cell lines. Figure 2B shows one parental cell line being treated with two chemotherapy agents, in two different doses or intervals producing four different daughter cell lines. By using a comparative development strategy, it is hoped that at least one of them will successfully produce a stable drug-resistant model. It can be interesting to observe which strategies produced resistance and which did not. These may be useful observations for the clinical treatment of cancer.

An example of a comparative selection strategy used H69 SCLC cells treated with cisplatin or oxaliplatin for two time periods, 2 h or 4 days. The 4-day pulse selection produced more stable resistance than the 2-h pulse (19) (Table 2). Had only the 2-h pulsed treatment been tested in this model drug resistance would have not been developed. The comparative nature of the selection also led to the finding that oxaliplatin resistance developed faster than cisplatin resistance in H69 cells (19).

A large study by Tegze et al. aimed to develop 40 drug-resistant models from MCF-7 and MDA-MB-231 breast cancer cells using doxorubicin and paclitaxel as selecting agents. They succeeded in making 29 drug-resistant models, 10 doxorubicin and 4 paclitaxel-resistant MCF-7 cell lines, and 6 doxorubicin and 9 paclitaxel-resistant MDA-MB-231 cell lines. From this study it appears that paclitaxel resistance was easier to develop in MDA-MB-231 (ER-negative) cells and doxorubicin resistance was easier to develop in MCF-7 (ER-positive) cells.

A study by our group (18) developed resistance to paclitaxel and carboplatin in large cell lung cancer cell lines (H1299 and H460). Cells at low confluence in 75cm^2^ flasks were exposed to 50 μg/mL carboplatin and 150 ng/mL (H1299) or 50 ng/mL (H460) paclitaxel for 4 h. After this period, the drug was removed and the flasks were rinsed and fed with fresh complete media. The cells were then grown in drug-free media for 6 days, replenishing the media every 2–3 days. This was repeated once a week for 10 weeks. Table 4 shows the IC_50_s of the platinum and taxane-resistant cell lines to a variety of chemotherapy agents. Resistance in the carboplatin-selected cells (1.5–2.3-fold) was considerably less than the resistance obtained in the paclitaxel-selected cells (2.4–4.4-fold). Selected cell lines show no obvious cross resistance pattern except within families of drugs, e.g., paclitaxel and docetaxel; carboplatin and cisplatin. The TAX-selected cells were also found to be resistant to vincristine, which is unsurprising since both agents affect microtubules. Both carboplatin-selected cell lines had a modest but statistically significant increased resistance to paclitaxel. The different patterns of resistance in cell lines selected under similar conditions show the complicated nature of multiple-drug resistance. For example, H1299-cpt became sensitive to vincristine, while after identical drug treatment, H460-cpt developed significant resistance to vincristine (2.9-fold). Overall, the low-level resistance (two- to five-fold) observed in these selected cell lines may be more clinically relevant to study than higher levels of resistance and this study highlights the importance to studying mechanisms in multiple models to identify relevant pathways.

**Table 4:** **Fold resistance of H1299 and H460 resistant variants compared with their parental cell lines**.

Chemotherapeutic agent	H1299-cpt	H1299-txl	H460-cpt	H460-txl
Carboplatin	2.0**	1.7***	2.3*	0.8*
Cisplatin	1.5*	1.5	1.6	0.7
5-FU	1.0	1.8**	0.9	1.1
VP-16	1.4**	1.1	0.9	1
Vincristine	0.8*	2.3*	2.9***	2.5
Adriamycin	0.9	1	1	0.9
Paclitaxel	1.2*	4.4***	1.6***	2.4***
Docetaxel	0.6	2.5***	2.3	2.8***

*p-Value <0.05; **p-value <0.01; ***p-value <0.005.

If a selection strategy fails to develop resistance, the treatment conditions can be altered in an attempt to produce higher levels of resistance. If the cells are growing very well after drug treatment, consider dose escalation. In some cases this may push the dose used above clinically relevant levels but it will increase the chance of resistance developing. Alternatively, the length of time the cells are exposed to drug can be increased or a pulsed-selection strategy could be converted to a continuous selection strategy. This may make the model less clinically relevant but may produce resistance that can be studied in the laboratory.

## Case Studies of Drug-Resistant Cell Lines

The following section presents two case studies of drug-resistant cell lines developed in our laboratory, the reasons that selection conditions were chosen and the drug resistance outcomes of the developed cell lines. A case study using continuous selection is presented for lapatinib in breast cancer. A case study using pulsed selection is presented for temozolomide-resistant melanoma.

### Lapatinib-resistant breast cancer cells – continuous selection

Of the published models of acquired lapatinib resistance there is very little commonality in the procedures used to condition the cells, in either the concentrations of lapatinib used or in the determination of resistance status (Table 5) (65–72). For instance, the procedures used to develop models of acquired lapatinib resistance included a single cell cloning technique (65, 68) fixed dose conditioning (69) and dose escalation conditioning (67, 71, 72). There was significant variation in the concentrations of lapatinib used to condition the cells; many studies began with a low dose of lapatinib (e.g., 100 nM) which was dose-escalated to upwards of 2 μM. Fixed concentration conditioning was performed with concentrations of lapatinib ranging from 3 to 10 μM. The length of conditioning required to achieve resistance varied from study to study with the majority of studies taking ~12 weeks to achieve resistance, whereas other studies took up to 1 year to achieve resistance. Another variation in different models of lapatinib resistance was the definition of lapatinib resistance. Most of the studies defined their conditioned cell lines as resistant based on their ability to grow in the presence of the concentration of lapatinib used to condition the cells, only one study used an IC_50_ method while a number of studies did not quantify the level of resistance. In contrast to the previously published models of acquired lapatinib resistance, the resistant models developed by us use a relatively low dose of lapatinib relative to the IC_50_ of the resulting cell line. To our knowledge our model of acquired lapatinib resistance, HCC1954-L are the first to show that extended exposure to low dose lapatinib results in significant lapatinib resistance, with resulting lapatinib IC_50_ values significantly higher than the concentration used for conditioning.

**Table 5:** **Published cell line models of acquired lapatinib resistance, the method and concentration used to condition the cells and the proposed mechanism of lapatinib resistance**.

Parent cell line	Conditioning method	Lapatinib concentration	Profiling technique	Resistance mechanism	Reference
BT474	Single cell cloning	5 μM^a^	Affymetrix array	Upregulation of ER signaling	(65)
BT474, SKBR3	Single cell cloning	5 μM^a^	Affymetrix array	Activation of RelA	(66)
SUM190	Continuous exposure	(0.25–2.5 μM)	Immunoblotting	Overexpression of XIAP	(67)
BT474	Single cell cloning	3 μM^a^	phospho-tyrosine immunoblotting	Overexpression of AXL	(68)
HCT116	Continuous exposure	10 μM^a^	Immunoblotting	Increased expression of MCL-1	(69)
HCC1954, BT474	Continuous exposure	(0.1–1 μM)	Immunoblotting	Increased expression of β1-integrin	(70)
SKBR3, MDA-MB-361, UACC893, BT474, HCC1954, SUM190	Continuous exposure	Increasing concentration up to 1 or 2 μM	Phospho-proteomic profiling	Increased SRC kinase activity	(71)
BT474, UACC812	Continuous exposure	(0.1–1 μM)	Immunoblotting	Upregulation of ER signaling	(72)

*^a^Denotes greater than peak-plasma concentration (2.5 μM)*.

**Figure 3:**
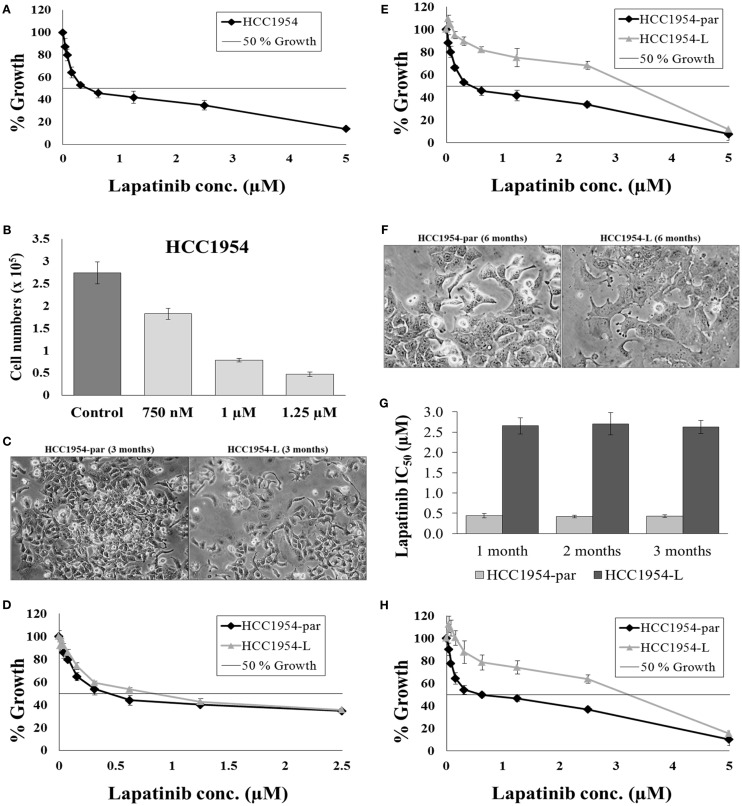
**Continuous selection of HCC1954 with lapatinib**. **(A)** Proliferation of HCC1954 cells following a 5-day treatment with lapatinib. **(B)** HCC1954 cells treated with varying concentrations of lapatinib over a 4-day period. Cell counts were performed using ViaCount reagent and Guava Software and expressed relative to control untreated cells. **(C)** Images of HCC1954-par and HCC1954-L cells after 3 months of lapatinib conditioning at 100× magnification. **(D)** After 3 months conditioning with 1 μM lapatinib, the proliferation of HCC1954-par and HCC1954-L cells was measured. **(E)** After 6 months conditioning with lapatinib, the proliferation of HCC1954-par and HCC1954-L was assessed. **(F)** Images of HCC1954-par and HCC1954-L cells after 6 months of lapatinib conditioning at 200× magnification. **(G)** Sensitivity of HCC1954-par and HCC1954-L cells to lapatinib following a freeze-thaw cycle. Growth is expressed relative to untreated control cells. **(H)** Lapatinib IC_50_ values for HCC1954-par and HCC1954-L cells following 1, 2, and 3 months growth in the absence of lapatinib. All growth rates and IC_50_s were calculated following a 5-day lapatinib treatment. Growth is expressed relative to untreated control cells. All error bars represent the standard deviation of triplicate experiments. Student’s *t*-test was performed to determine significant differences: *p < 0.05; **p < 0.01.

HCC1954 cells overexpress HER2 (73) and therefore represent a cell line model of HER2-positive breast cancer. Lapatinib is a tyrosine kinase inhibitor that targets the intracellular domain of HER2 and EGFR and is approved for the treatment of HER2-positive breast cancer (74, 75). HCC1954 are moderately sensitive to lapatinib with an IC_50_ of 0.43 ± 0.03 μM (Figure 3A). Lapatinib is administered to cancer patients orally with a dose of 1000–1250 mg given daily (76). The median peak-plasma concentration of lapatinib reported in patients receiving 1200 mg lapatinib (once daily) was 1.2 μg/mL (2.1 μM) and the median steady-state trough concentration was 0.3 μg/mL (0.5 μM), with a range of 0.2–0.5 μg/mL (77). Therefore a continuous selection strategy is clinically relevant for lapatinib. To optimize the dose of drug used for selection, a lapatinib dose response assay was performed in order to determine the concentration of lapatinib which would result in 70% growth inhibition over a 4-day treatment. Treatment of HCC1954 cells with 1 μM lapatinib inhibited the growth of the cells by 71.5 ± 1.2% compared to untreated controls (*p* = 0.004) (Figure 3B). Therefore a selection strategy of continuous exposure of HCC1954 to 1 μM lapatinib was initiated with the media replenished every 4 days with fresh drug. The selection strategy was conducted in duplicate with “A” and “B” flasks as a backup in case there were problems with one flask.

HCC1954 cells were seeded into two flasks; 1 × 10^6^ cells per 75cm^2^ flask. One flask was left untreated but was passaged alongside the treatment flask and named HCC1954-par, the cells treated with 1 μM lapatinib were named HCC1954-L. It is important to passage the untreated parental cells alongside the treated cells as a control as continuous cell culture can result in alterations in cellular characteristics, including drug resistance. The morphology of both cell lines and the sensitivity of the cell lines to lapatinib were monitored throughout the selection. After 3 months of treatment, the morphology of HCC1954-L was not altered (Figure 3C). For all cytotoxicity assays the HCC1954-L cells were grown in drug-free media for 5 days prior to testing. The lapatinib IC_50_ value for the HCC1954-par cells was 0.42 ± 0.01 μM, which is similar to the original HCC1954 cells. The lapatinib IC_50_ value for the HCC1954-L cells was 0.75 ± 0.07 μM (Figure 3D). This represents 1.8-fold increase in resistance to lapatinib. At this stage of the treatment process the lapatinib IC_50_ of HCC1954-L cells had not yet exceeded the treatment dose however they had begun to actively proliferate in the presence of lapatinib. The concentration of lapatinib was therefore increased from 1 to 1.25 μM and conditioning continued with this concentration for a further 3 months.

After 6 months of lapatinib conditioning, the sensitivity of the cells was again tested. Both the “A” and “B” flasks of HCC1954-L cells developed equivalent amounts of resistance, and the “As” were chosen for all subsequent experiments and the “Bs” frozen as a backup. The lapatinib IC_50_ value for the HCC1954-par cells was 0.42 ± 0.02 μM whereas the lapatinib IC_50_ for HCC1954-L cells was 2.67 ± 0.08 μM (*p* = 0.01) (Figure 3E). This represents 6.1-fold increase in resistance to lapatinib. HCC1954-L cells were deemed to be resistant to lapatinib as the lapatinib IC_50_ was above the 1 μM threshold for lapatinib sensitivity (78). The resistant cells also exhibited distinct morphological alterations compared to the parental cell line. These differences were indicated by more distinct colony boundaries and a flatter cell shape (Figure 3F).

In order to assess the stability of acquired resistance in the HCC1954-L cell line, sensitivity to lapatinib was assessed after freezing and thawing and following drug withdrawal. To establish a reliable cell line model of lapatinib resistance the phenotype must be stable when the cell line is frozen and re-thawed. To assess this, frozen stocks of the HCC1954-par and HCC1954-L cells were prepared in fetal calf serum containing 5% DMSO. After a minimum of 48 h in liquid nitrogen the frozen stocks were thawed and the viability of the stocks assessed by microscopy. The cells were then passaged a minimum of 3 times before lapatinib sensitivity assays were repeated (Figure 3G). The lapatinib IC_50_ was 0.44 ± 0.02 μM in the parental cells while the lapatinib IC_50_ in HCC1954-L cells was 2.73 ± 0.05 μM. This indicates that the HCC1954-L cells retain their resistant phenotype following a freeze/thaw cycle.

In order to access the long-term stability of the resistant phenotype, drug withdrawal assays were performed. Lapatinib was removed from the HCC1954-L cells and the sensitivity of the cells to lapatinib was tested at 4-week intervals for a period of 12 weeks, the results at each interval are illustrated in (Figure 3G). Following 12 weeks growth in the absence of lapatinib the lapatinib IC_50_ of the parental cells was 0.43 ± 0.05 μM while the lapatinib IC_50_ of HCC1954-L cells was 2.63 ± 0.16 μM. There was no significant difference between the initial lapatinib IC50 for either the parental or resistant cell line and the lapatinib IC50 for the cell lines after 12 weeks growth in the absence of lapatinib (Figure 3H).

Therefore, we successfully established a stable cell line of acquired lapatinib resistance (HCC1954-L) induced by long-term continuous treatment with sub-peak-plasma concentrations of lapatinib. The mechanisms of acquired resistance to lapatinib are being investigated in this model using proteomics and genomic techniques.

### Temozolomide-resistant melanoma cell lines – pulsed selection

Temozolomide is frequently used to treat metastatic melanoma. No dosing schedule of temozolomide has been clinically proven to be more effective than a single administration of temozolomide (79); however current treatments favor a 5-day treatment schedule (80). We found that the IC_50_ concentrations of temozolomide were in the high micromolar range in melanoma cell lines. Previous studies in two melanoma cell lines demonstrated temozolomide IC_50_ concentrations of ~800 μM (81), which is consistent with the values observed in our cell line panel of six melanoma cell lines (temozolomide IC_50_ ranged from 250 to 800 μM). However, in the clinical setting plasma levels of temozolomide only reach concentrations approaching 80 μM (82) The half-life of temozolomide is <2 h (83), which would reduce the efficacy of the drug in patients and may also explain the high IC_50_ values observed *in vitro*. A pulsed selection of drug was chosen to mimic these pharmacokinetic properties of temozolomide.

**Figure 4:**
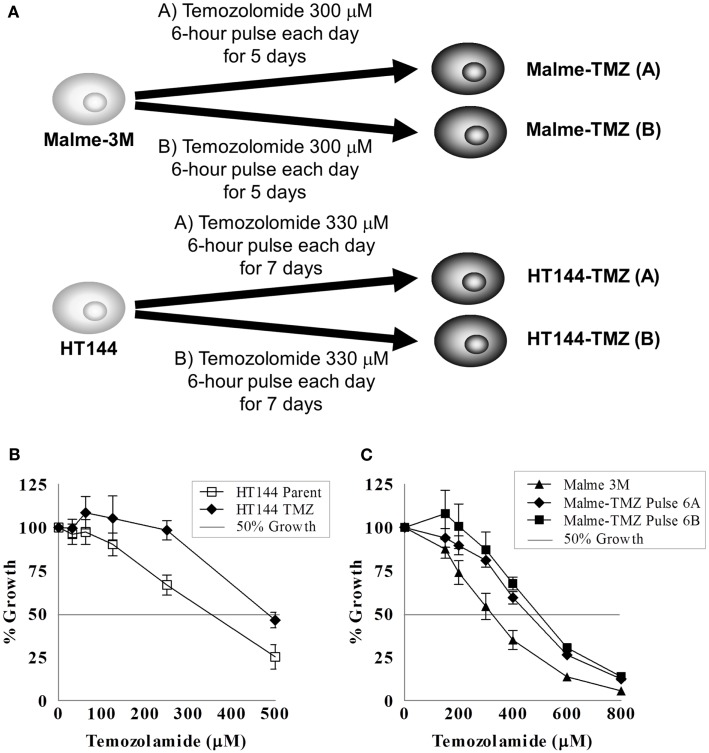
**Pulse selection of Malme-3M and HT144 with temozolomide**. **(A)** Selection strategy of Malme-3M and HT144, each treatment of temozolomide was performed in duplicate. Effect of temozolomide in Malme-3M and HT144 and temozolomide “pulse selected” resistant variants. **(B)** Malme-TMZ(A) and Maleme-TMZ(B) vs. Malme-3M cells **(C)**. HT144-TMZ vs. HT144 cells. Error bars represent the standard deviation of triplicate assays.

Malme-3M or HT144 melanoma cell lines were seeded at a density of 2.5 × 10^4^ cells in a 75 cm^2^ flask. The entire selection strategy was conducted in duplicate; two flasks of each cell line were set up for untreated control flasks and two for temozolomide selection (Figure 4A). Cells were allowed to attach for 24 h prior to treatment with chemotherapy. For Malme-3M cells, after each treatment cells were allowed to grow until confluent, then trypsinised and reseeded at a density of 2.5 × 10^4^ cells per flask for the next round of selection. For HT144 cells, cells were grown in the flask for the 5 days of their treatment, then left to grow to confluence. After cells recovered they were trypsinised reseeded at 2.5 × 10^4^ cells per flask for the next round of selection.

The Malme-3M cells were pulse treated with 300 μM temozolomide for 6 h and then the drug containing medium removed and replaced with fresh drug-free medium. This single pulse treatment was repeated six times. HT144 cells were treated for 6 h daily with 330 μM temozolomide for 5 days. After the five daily treatments the drug was removed and replaced with fresh drug-free media. This treatment was repeated four times. This treatment schedule was used to replicate that of the clinical setting, where temozolomide is administered daily for 5 days, followed by a period of no treatment (84). The pulse selection strategy used in these cells allowed us to compare differences between the clinical daily administration and the lab based pulse selection to observe if either regimen resulted in increased levels of resistance acquired.

The IC_50_ for temozolomide in Malme-3M parent cells is 306 ± 29 μM. Malme-TMZ(A) and Malme-TMZ(B) display significantly increased IC_50_s for temozolomide of 440 ± 21 μM [1.44-fold increase (*p* = 0.004)] and 515 ± 45 μM [1.68-fold increase (*p* = 0.04)] (Figure 4B). The IC_50_ for temozolomide in HT144 cells is 338 ± 25 μM. In HT144-TMZ(A), the pulse-selected variant of HT144, the IC_50_ increased to 490 ± 15 μM, which represents a 1.45-fold increase in resistance to TMZ (*p* = 0.002) (Figure 4C). HT144(B) did not develop significant resistance to temozolomide, and so was not used in further studies.

During drug selection of cell lines, cells can acquire altered sensitivity to other chemotherapeutic drugs. The two temozolomide-selected cell lines from each parent cell line with the highest levels of resistance [Malme-TMZ(B) and HT144(A)] were tested with four drugs to examine the chemosensitivity between the parent and the resistant cell lines (Table 6). The melanoma cell line HT144 and the temozolomide-selected variant HT144-TMZ display similar sensitivity to cisplatin and epirubicin whilst the resistant cell line is significantly more sensitive to mitoxantrone (*p* = 0.02). Malme-3M and the pulse-selected cell line Malme-TMZ have similar IC_50_s for EPI and mitoxantrone. Malme-TMZ is significantly more resistant to cisplatin (*p* = 0.001) and both HT144-TMZ and Malme-TMZ are significantly more resistant to docetaxel than the parent cell lines Malme-3M and HT144 (*p* = 0.02; *p* = 0.02), although the IC_50_ values are still in the very low nanomolar range.

**Table 6:** **Fold resistance of HT144 and Malme-3M resistant variants compared with their parental cell lines**.

Chemotherapeutic agent	HT144-TMZ	Malme-TMZ
Cisplatin (nM)	1.4	2.0*
Epirubicin (nM)	1.3	0.8
Mitoxantrone (nM)	0.2*	1.3
Docetaxel (nM)	1.4*	1.2*

*Indicates a *p*-value <0.05 as calculated by Student’s *t*-test.

Two temozolomide-resistant cell lines [Malme-TMZ(B) and HT144(A)] were established using two different selection methods. Duplicate selection proved useful in this selection strategy as one of the variants HT144-TMZ(B) did not develop resistance. Although the level of resistance induced was relatively low, these two cell lines provide unique clinically relevant models to study acquired temozolomide resistance in melanoma (85). The temozolomide-resistant variants were cross resistant to cisplatin. As temozolomide and cisplatin are both DNA damaging agents, there may be common mechanisms of resistance to the DNA damage induced by these agents.

## Maintaining Drug-Resistant Cell Lines for Research

Once resistance has been established with the selection strategy the stability of the resistance needs to be determined. One important test of the stability of the model is the recovery of the drug-resistant phenotype from the frozen stocks. If the phenotype is lost or resistance is significantly lower on freeze thaw then the model will not be practical to use in the laboratory. If the resistance is not stable on freeze thaw then the drug-resistant cells need to be treated for longer, possibly with a higher dose of chemotherapeutic.

**Figure 5:**
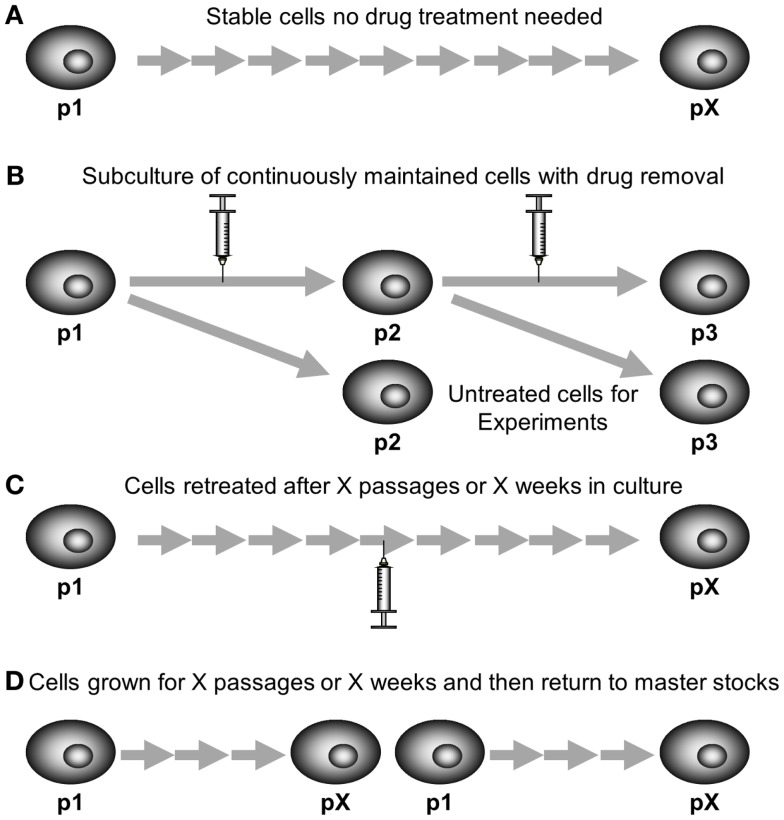
**Maintaining drug-resistant cell lines in cell culture**. **(A)** Stable cell lines require no drug treatment to maintain their resistant phenotype. **(B)** Some cell lines are grown continually in the presence of chemotherapy, chemotherapy needs to be removed for one subculture prior to using for experiments. **(C)** Some models are repeat pulse treated after a certain number of passages or weeks in culture once their resistant phenotype begins to fade. **(D)** Some models are discarded and new cells grown from master stocks after a certain number of passages or weeks in culture once their resistant phenotype begins to fade.

The long-term stability of resistance also needs to be examined. Resistant cell models that have been selected by continuous exposure to drug should be grown for several months to determine if the resistance phenotype remains present. Some cell lines may be completely stably resistant (Figure 5A) and are grown in the absence of chemotherapeutic, such as DLKP-A or IGROVCDDP (21, 25). Regular monitoring by cytotoxicity assay is required to make sure that the resistance phenotype of the cell lines persists.

Alternatively, the cells can be grown continuously in chemotherapeutic, either at the dose used in selection or a lower maintenance dose. This may be if the cells are not stably resistant on removal of the chemotherapeutic or if the researchers wish to ensure consistency of experiments. KB-CP20 cisplatin-resistant cells which were selected with increasing concentrations of cisplatin up to 20 μg/mL over a period of 6 months. The resistance was then maintained in media containing 5 μg/mL cisplatin (22). KB-5-8-11 colchicine-resistant cells are also maintained in 100 ng/mL of colchicine (24). Resistant cell models which are maintained in chemotherapy drug need to be grown in drug-free media for a passage prior to conducting experiments (86). This is so that the drug-free controls of experiments are cells not exposed to drug, rather than cells grown in the maintenance dose of chemotherapy. Figure 5B shows a subculture schematic for this technique. Another approach to maintaining stability is growing cells in drug-free media but using a pulse treatment at regular intervals (Figure 5C). This can be used even if the cell model was originally developed by continuous exposure. The CEM/E25 and CEM/E1000 epirubicin-resistant variants of CCRF-CEM leukemia cells were established by continuous exposure then grown without drug and resistance was maintained by repeat pulse treatment every 6 weeks with the selecting doses of epirubicin 25 and 1000 ng/mL, respectively (15, 16).

Resistant models that are selected by pulse selection are often less stable than their continuously selected counterparts. However, IGROVCDDP is a stably resistant cell line established by pulse selection using dose escalation (21). Pulse-selected cell lines which lose their resistant phenotype can also be maintained by re-treatment with the selecting dose (Figure 5C) such as K562/DNR resistant leukemia cells (17). Alternatively, instead of repeating the pulse treatment, resistant cells can be grown for a certain number of weeks or passages and then new stocks are defrosted of an earlier passage with the resistant phenotype present (Figure 5D). H69CIS200 and H69OX400 cisplatin and oxaliplatin-resistant cells were 1.5–2-fold resistant to platinums for 5–6 weeks in drug-free culture and then the resistance phenotype faded over the next 6–8 weeks in culture (20). This technique is also often used with resistant models regardless of selection strategy to ensure consistency, so that cells within a limited range of passage numbers are used for all experiments.

## A Reproducible Experiment?

Many mechanisms of resistance exist for each chemotherapy drug. The chemotherapeutic drug cisplatin has been studied in drug-resistant cell models for many years and mechanisms of resistance include decreased accumulation of drug, inactivation by glutathione and increased DNA repair (87). These mechanisms need not all occur in the same drug-resistant model. Over time more common mechanisms will be identified by their occurrence in many drug-resistant models.

A comparative selection strategy could involve parallel selections of the same parental cell line with the same chemotherapy agent, under the same treatment conditions. Similar or different mechanisms could develop in these independent treatments. This is the randomness of natural selection. A study by Tegze et al. developed multiple drug-resistant cell lines from MCF-7 and MDA-MB-231 breast cancer cells (88). The parent cell lines were split and new cell lines were generated in parallel by treatment with gradually increasing concentration of doxorubicin or paclitaxel. The study aimed to produce 10 resistant sublines for each agent in each parent cell line. Using a continuous treatment strategy they produced 29 resistant models over an 18-month period. There were 10 doxorubicin and 4 paclitaxel-resistant MCF-7 cell lines and 6 doxorubicin and 9 paclitaxel-resistant MDA-MB-231 cell lines. The fold resistance values compared to the parental cell lines show up to 46- and 28-fold resistance to doxorubicin and paclitaxel, respectively. The cell lines turned out to be highly heterogeneous for the mechanisms of drug resistance present, and in general only a few mechanisms are activated in one cell line to achieve drug resistance. Of note, the expression of P-gp did not correlate with resistance in the cell line models, despite the development of models with two P-gp substrates. This suggests that in some of the models P-gp was activated early in the selection process and became a dominant mechanism, in others this did not occur.

Two models of cisplatin resistance were developed from H69 SCLC cells in the same research group in successive years (19, 89). These models were developed independently rather than in parallel. H69-CP and H69CIS200 were developed with 100 or 200 ng/mL of cisplatin, respectively. Both cell models were two- to four-fold resistant to cisplatin, and had decreased expression of p21 which may increase the cell’s ability to progress through the cell cycle in the presence of DNA damage. Both the H69-CP and H69CIS200 cells showed no decrease in cellular cisplatin accumulation. However, the H69-CP cells have increased levels of cellular glutathione and are cross resistant to radiation whereas the H69CIS200 cells have neither of these changes.

The cell line IGROV-1 has been used to develop cisplatin drug-resistant models by many research groups. IGROVCDDP cisplatin-resistant cells have an unusual resistant phenotype; they are cross resistant to paclitaxel as they overexpress P-gp (90). It is unusual but not unprecedented to see a model of acquired cisplatin resistance overexpress P-gp (63, 91–94). This most likely represents a generalized stress response to long-term cisplatin treatment as cisplatin is not a P-gp substrate (95). IGROVCDDP cells do not have increased total cellular glutathione but the way glutathione is recycled within and from outside the cell is enhanced, increased enzyme activity of glutathione reductase and gamma-glutamyltransferase 1 (GGT1) was present (90). In contrast, IGROV-1/Pt0.5 and IGROV-1/Pt1, platinum-resistant cell lines are sensitive to P-gp substrates, have increased cellular glutathione and decreased GGT1 (96) which is the reverse pattern to that seen in the IGROVCDDP platinum/taxane-resistant cells. However, it should be noted, that different research groups can of course have different sub clones of a parent cell line and this can be a factor for the differences in the resistant models produced.

These examples demonstrate that the same cell line, treated with the same chemotherapy agent leads to the development of a heterogeneous range of drug-resistant models. Therefore, the development of drug-resistant models should be regarded as a process rather than an experiment that can be repeated in biological triplicate. If parallel models of the same treatment are produced the heterogeneity between drug-resistant cell lines should be examined with interest rather than dismissed as a non-reproducible experiment.

## Conclusion

We have provided a detailed guide to the decision-making process for the development and ongoing maintenance of drug-resistant cancer cell lines. There is no one right way to make drug-resistant cell lines. The case studies from our laboratories highlight how we have successfully developed models in a variety of ways for use in research projects.

## Methods

### Cell culture

H1299, H460, HCC1954, Malme-3M, OVCAR8, UPN251, and cells and their drug-resistant variants were grown in antibiotic and chemotherapy-free RPMI (Sigma #R8758). HT144 cells and their resistant variants were grown in antibiotic and chemotherapy-free McCoys 5A medium (Sigma). HT144, HCC1954, Malme-3M, OVCAR8, and UPN251 and their resistant variants were supplemented with 10% FCS (Lonza, Belgium). H1299, H460, and their resistant variants were supplemented with 5% FCS. All cell lines were maintained in a humidified atmosphere with 5% CO2 at 37°C. All cultures were tested routinely and were *mycoplasma-*free.

### Growth curves for optimization of selection doses

OVCAR8 or UPN251 cells were plated in duplicate into 6-well plates at a cell density of 1 × 10^4^ cells/mL in 1 mL media. A control plate was set up separately with duplicate wells. On day 2 1 mL of media with drug was added to all plates excluding the control, which received drug-free media to the same volume. On day 5 media was changed on all plates and replaced with drug-free media. The control plate and one drugged plate were taken down and cell counted. Cell counts for the control were compared to the drug treatment. A percentage cell survival was calculated in order to see the effects of drug treatment on cell growth/survival.

$$\begin{array}{llll} & \text{Percentage cell survival}\; & & \;\\ & \;=\frac{\text{Average cell number of drugged cells}}{\text{Average control cell number}}\times 100\; & & \; \end{array}$$

Over subsequent days one plate for each drug dose was observed under a light microscope to see when normal growth had returned. When cells were deemed to have returned to confluence this plate was cell counted to confirm recovery. Percentage cell survival will now be above or climbing to 100%. The time taken for cells to resume growth and return to confluence was recorded.

### Cytotoxicity assays

To determine the cytotoxicity of chemotherapy drugs, cell growth/viability was measured using an acid phosphatase assay; 1.5–3 × 10^3^ cells were seeded in flat-bottomed 96-well plates and incubated overnight prior to addition of drug. Chemotherapeutics were obtained from St Vincent’s University Hospital, Dublin, Ireland. Lapatinib was purchased from Sequoia. Temozolomide was obtained from the National Cancer Institute. Other inhibitors and modulators were obtained from Sigma. Drug-free controls were included in each assay. Plates were incubated for a further 5 (HCC1954, Malme-3M and HT144) or 7 days (H1299 and H460) at 37°C in a humidified atmosphere with 5% CO_2_ and cell viability was determined using an acid phosphatase assay (97). Growth of drug-treated cells was calculated relative to control untreated cells in biological triplicate.

### Statistics

All experiments were performed at minimum in triplicate. Two-sample, two-tailed Student’s *t*-tests were used to determine significant differences using *p* < 0.05 as a cut off.

## Author Contributions

Britta Stordal conceived the need for a review of methods of development of drug-resistant cell lines and wrote the manuscript. Martina McDermott, Alex J. Eustace, Steven Busschots, Laura Breen contributed their expertise in drug-resistant cell line development, contributed data for case studies and assisted with locating references, and drafting the manuscript. Norma O’Donovan, John Crown and Martin Clynes mentored the development of the drug-resistant cell lines used in the case studies, as well as contributing their expertise in drug-resistant cell line development. All authors approved the final version of the manuscript.

## Conflict of Interest Statement

The authors declare that the research was conducted in the absence of any commercial or financial relationships that could be construed as a potential conflict of interest.

## Abbreviations

5-FU, fluorouracil; ADR, adriamycin; AUC, area under concentration time curve; CHL, chlorambucil; CIS, cisplatin; CR, complete response; CSC, cancer stem cells; CYC, cyclophosphamide; DNR, daunorubicin; DOCE, docetaxel; DOX, doxorubicin; EPI, epirubicin; ETO, etoposide; GFR, glomerular filtration rate; IC_50_, 50% inhibitory concentration; IFO, ifosfamide; IV, intravenous; MEL, melphalan; MTT, 3-(4,5-dimethylthiazol-2-yl)-2,5-diphenyltetrazolium bromide; NR, no response; OX, oxaliplatin; PARP, poly (ADP-ribose) polymerase; PD, progressive disease; P-gp, P-glycoprotein; PR, partial response; PRED, prednimustine; RAD, radiation; SCLC, small cell lung cancer; TAX, paclitaxel; THI, thiotepa; UNK, unknown; VINC, vincristine; VIND, vindesine.
